# Generation of Domains for the Equine Musculoskeletal Rehabilitation Outcome Score: Development by Expert Consensus

**DOI:** 10.3390/ani10020203

**Published:** 2020-01-25

**Authors:** Gillian Tabor, Kathryn Nankervis, John Fernandes, Jane Williams

**Affiliations:** Equestrian Performance Research Group, Hartpury University, Gloucester GL19 3BE, UK; Kathryn.Nankervis@hartpury.ac.uk (K.N.); john.fernandes@hartpury.ac.uk (J.F.); jane.williams@hartpury.ac.uk (J.W.)

**Keywords:** equine, physiotherapy, outcome measures, rehabilitation, Delphi method

## Abstract

**Simple Summary:**

Within rehabilitation, measurements taken before, during and after treatments are used to judge patient progress and the effectiveness of prescribed treatments. To know which measurements to use for a given health condition, practitioners must have knowledge of what should be measured, which measurement tools are available and accurate, alongside what they intend to measure. Composite outcome measures (OMs) are tools which use grouped measurement tests to monitor patient progress; they have been tested for a variety of human and canine conditions but none have been designed or tested for use in physical rehabilitation in horses. This study asked leading equine veterinarians, physiotherapists and researchers which measures should be included in an OM for use in the rehabilitation of horses. Using a process to evaluate agreement, ten areas of measurement were included in the final model: lameness, pain at rest, pain during exercise, behaviour during exercise, muscular symmetry, performance/functional capacity, behaviour at rest, palpation, balance and proprioception. Existing reliable tests used to measure these areas were evaluated and potential new measures discussed and now should be taken forward to testing as a composite outcome score to see if they are effective in measuring effectiveness of treatment.

**Abstract:**

Outcome measures (OMs) are a requirement of professional practice standards in human and canine physiotherapy practice for measurement of health status. Measures such as pain and functional capacity of specific regions are used to track treatment impact and can be used to develop optimal management strategies. To achieve comparable patient care in equine physiotherapy, OMs must be incorporated into practice; however, no reliable and valid OMs exist for equine rehabilitation. This study utilised the experience and opinion of a panel of experts working in the equine rehabilitation sphere to gain consensus on the core areas (domains) to be included in a model, to lead to an OM scale for horses undergoing rehabilitation. The Delphi method and content validity ratio testing was used to determine agreement with domains reaching the critical value required for inclusion. The expert panel agreed on ten domains to be included in the OM scale: lameness, pain at rest, pain during exercise, behaviour during exercise, muscular symmetry, performance/functional capacity, behaviour at rest, palpation, balance and proprioception. An OM with these domains would provide a holistic objective assessment tool which could be used by equine rehabilitation professionals in clinical practice.

## 1. Introduction

Physiotherapy is recommended for a number of equine musculoskeletal conditions such as overriding dorsal spinous processes and thoracolumbosacral pain, soft tissue injuries such as ligament and tendon injuries and osteoarthritis [1,2]. However, the degree of detail regarding the specific physiotherapy interventions, such as for treatments including manual therapy [3], electrotherapy [1] or exercise therapy [4,5], either individually or in combination, varies between publications ranging from trials, often with low subject numbers to clinical review papers. As a result, equine physiotherapists use this information in combination with their experience and clinical reasoning to select treatment interventions they consider effective [6]. For example, in rehabilitation plans for overriding dorsal spinous processes, exercises to encourage ventral flexion to separate the spinous processes [7] are used in combination with exercises to strengthen the deeper ‘core’ stability muscle *multifidus* [8]. For thoracolumbosacral pain, electrotherapy in the form of neuromuscular electrical stimulation [7,9] or manual therapy [10,11] are commonly applied. However due to variation in practitioners experience, and the distinct nature of each patient, there are no standardised practice guidelines for equine rehabilitation. This lack of standardisation places increased emphasis on the physiotherapists’ ability to assess each horse’s progress to ensure they meet their duty of care to the patient despite the currently limited evidence base to support this decision making [12]. 

A common feature in published studies that include physiotherapy techniques is a lack of objectivity when reporting on the outcome (where outcome is defined as ‘*any identified result arising from exposure to a causal factor or a health intervention*’) [13]. Within human orthopaedic research, Chiarotto et al. [14] suggested that outcomes are inconsistently measured and reported across trials of health interventions for low back pain in humans [14]. Similarly, in equine research subjective outcomes (e.g., decisions on success based on horse-owner survey) are reported after surgery for over-riding dorsal spinous processes [7,15,16] and the treatment of sacro-iliac disease [17]. The lack of outcome measurement reduces the ability to compare findings between studies and potentially encourages selective reporting of favourable outcomes [14]. This will impact ongoing practice and *may* result in confirmation bias when assessing subsequent outcomes, thus placing the patient at risk of lack of progress, or worse still, deterioration of their situation. Given the duty of care that a physiotherapist has with their patient, this remains an important issue. In human research, to reduce the heterogeneity of outcome measures (OMs) in clinical trials, a minimum sets of outcomes that should be measured by clinicians and reported for a particular health condition have been agreed upon [14]. These specific measurement tools or techniques are known as outcome measures and a grouping of OMs can be used to form a composite outcome score that can then be used to assess the short- and long-term effect of rehabilitation for the patient [18].

OMs have been developed for use in human practice for the measurement of health status and include measures of pain and functional capacity in specific regions, used to track impact of treatment and thus, the development of optimal management strategies [19]. For sport injuries, the Victorian Institute of Sport Assessment Scales for patella tendinosis and achilles tendinopathy, and the Copenhagen Hip and Groin Outcome Score are examples of OMs that have been generated to score pain, symptoms and physical function [20,21,22]. For dogs, outcomes can be measured with the Helsinki Chronic Pain Index, the Canine Brief Pain Index or the Finnish neurological function testing battery for dogs named the FINFUN [23,24,25]. These examples of composite OMs for humans and dogs have face validity, have undergone reliability and validity testing, and are used in clinical practice; however, no composite OMs have been developed for equine physical rehabilitation.

To achieve comparable professional practice standards in equine physiotherapy, OMs must be incorporated into practice [6]. To date, a few equine specific OMs that measure a single factor in clinical practice (referred to as objective markers (OBJM)) have been subject to reliability testing but there are no composite equine OMs. OBJMs include the use of pressure algometry [26,27], manual palpation scoring [28,29], posture/muscle size measurement from photographs [30], muscle dimension measurement using a flexicurve ruler [31], range of joint motion using a universal goniometer [32,33] and evaluation of pain-related behaviour [34]. Despite these studies, the use of OBJM in clinical practice is sparse and clinicians report this being due to the lack of available, validated and reliable OBJMs [6], suggesting a lack of awareness to the available evidence. In a recent survey, equine physiotherapists stressed that OBJMs and OMs need to be simple to use, inexpensive and relevant to the cases they see [6]. It is unknown which domains clinicians working in the equine rehabilitation industry would consider valuable to measure and how these could be combined to generate a composite outcome score specific and relevant for the cases practitioners work with. The aim of this study was to determine which domains should be measured within equine musculoskeletal rehabilitation to develop a globally useful composite outcome score.

## 2. Materials and Methods 

The methodology was guided by international best practice guidelines for the development of patient reported outcome measures [35] and involved iterative stages using a mixed methods approach that involved a literature review [36] and expert input. The Delphi method of gathering data was used to gain a convergence of opinion from the invited selection of veterinarians, physiotherapists and equine researchers located world-wide. The Delphi method, which is an accepted method for achieving convergence of opinion, was selected as a technique using group communication from a panel of experts [37]. Using this method, the panel members are able to review and revise their responses in the stages of the process [38] and the controlled feedback process provides anonymity to the respondents, which may be a factor in group-based discussions [37].

### 2.1. Delphi Step 1

Via email, 35 subject matter specialists, based in Europe and the United States were invited to participate in the study based on their expertise in equine rehabilitation. These included ten equine veterinary surgeons with greater than 10 years clinical experience, all of whom are published in equine musculoskeletal health and behaviour research; fifteen UK Chartered Physiotherapists (Association of Chartered Physiotherapists in Animal Therapy, category A members) with greater than 10 years equine practice experience; and ten equine research professionals, with an interest in equine musculoskeletal rehabilitation and performance working in equine higher education institutes. Consent by participants, to be included in the Delphi process, was gained via response to the first email in step one, which also confirmed responses would be compiled anonymously.

Once invited to participate each expert was asked to reply with confirmation that they wished to be included in further rounds of the process and asked to suggest domains to be included. The term domain was defined as an area of measurement that could be included within an OM for equine musculoskeletal rehabilitation.

At this stage, the number of survey rounds was not fixed and was to be determined by the degree of consensus within the panel of experts. We did, however, expect there to be between three and five rounds with the last providing a final opportunity for the experts to revise their judgments [37]. 

### 2.2. Delphi Step 2

An email with a link to a questionnaire (SurveyMonkey, San Mateo, CA, USA) was sent out to the experts that responded positively to being included in the Delphi panel. This stage was designed to assist selection of the domains that should be included in the final tool termed ‘the equine musculoskeletal rehabilitation outcome score (TEMROS)’ with the option to suggest other areas that could also be included. There was potentially a large range of domains that could be part of the outcome score; thus, to keep the outcome score practitioner friendly, valid and reliable, the number of domains included needed to be limited by consensus of the Delphi panel. The experts were provided with a list of domains collated from the response of the first email round. Within the second questionnaire, each domain required the expert to mark whether the specific outcome was essential, useful but not essential, not useful or if the expert was unsure if it should be included as an area of measurement for the purpose of musculoskeletal assessment in a horse undergoing rehabilitation [39,40]. 

### 2.3. Delphi Step 3

From the responses gained, a content validation process was used to agree to include or discard items listed as possible domains (Lawshe, 1975) with content validity ratio (CVR) and critical values used to confirm the level of agreement that exceeds that of chance [40].

(1) $$\mathrm{CVR}=\frac{n_{e}-(N/2)}{N/2}$$

where CVR is content validity ratio, n_e_ is the number of essential members and N is the number of panel members [39].

Perfect agreement would result in +1 and perfect disagreement results in a CVR of −1. This process was used to identify the domains to be included in TEMROS.

### 2.4. Delphi Step 4 

The list of domains that met the agreement criteria were emailed to the panel of experts who were invited to comment on the final selection. 

## 3. Results

### 3.1. Delphi Step 1 

Seven veterinary surgeons, eleven ACPAT Physiotherapists and six equine industry experts agreed to be included in the Dephi process and fifteen potential domains were suggested. These fifteen domains were taken forwards to the questionnaire in step 2.

### 3.2. Delphi Step 2

**Figure 1:**
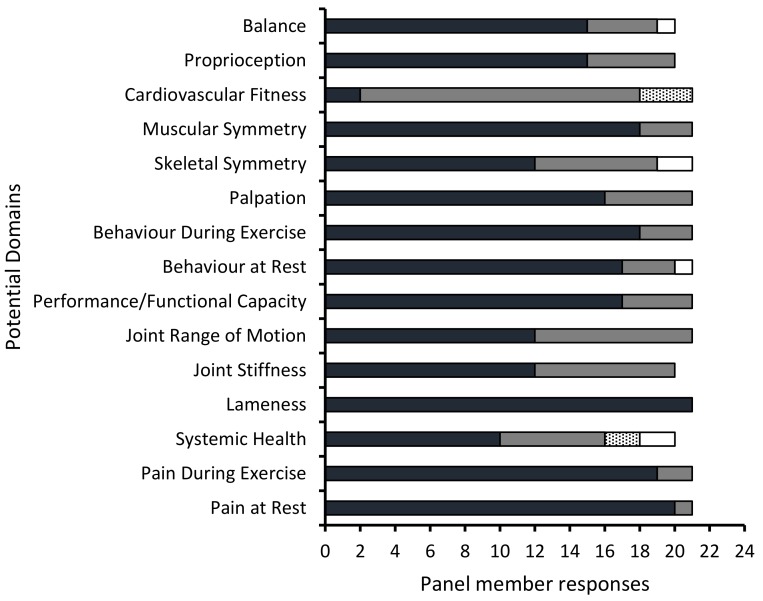
Expert opinion on domains to be included in an equine musculoskeletal outcome score.

The questionnaire was returned by 21 of the 24 experts from step 1 and the data tabulated (Figure 1).

### 3.3. Delphi Step 3

The critical number required for the proportion in agreement (considering the domain to be essential) for a panel of 21 members according to Ayre and Scally [40] is 15 (71.4%), with a minimum CVR critical value of 0.429 [40]. Therefore, using content validity ratios, the number of possible domains for inclusion in TEMROS was reduced from 16 to 10. These were, with CVR values provided in parentheses: lameness (1.00), pain at rest (0.91), pain during exercise (0.81), behaviour during exercise (0.71), muscular symmetry (0.71), performance/functional capacity (0.62), behaviour at rest (0.62), palpation (0.52), balance (0.50) and proprioception (0.50). The domains with CVR critical values less than the required critical value were: joint stiffness (0.20), joint range of movement (0.14), skeletal symmetry (0.14), systemic health (0.00) and cardiovascular fitness (−0.81).

### 3.4. Delphi Step 4

Seven panel members responded to the list of 10 domains positively and there were no further domains proposed for inclusion. There were three comments that centered on domains that should not be included. Three experts suggested that systemic health does not need to be measured within an outcome score, as this should be a pre-requisite for undertaking a rehabilitation programme and two mentioned cardio-vascular fitness measurement being outside the scope of a musculoskeletal assessment tool.

## 4. Discussion

**Figure 2:**
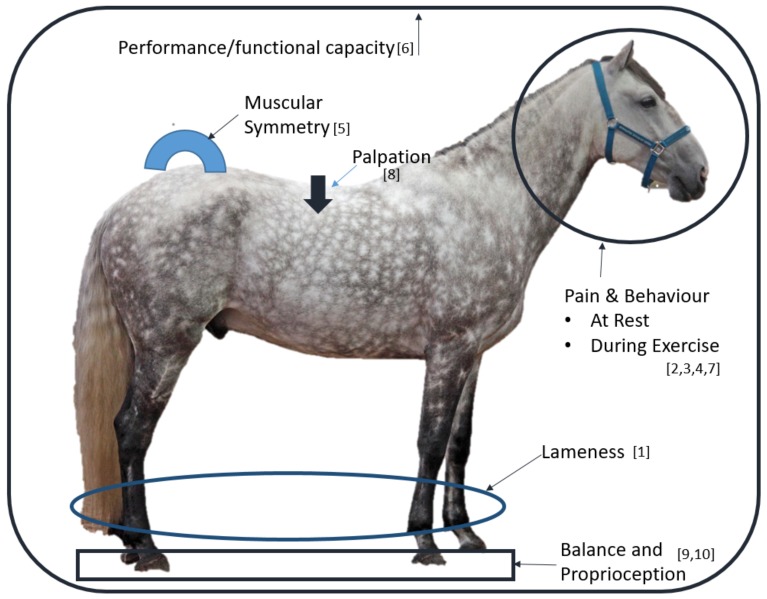
Ten domains for measurement, as agreed by the expert panel, to be included in outcome score for equine musculoskeletal rehabilitation. In order of highest agreement the domains (with number in square brackets) are: 1: lameness, 2: pain at rest, 3: pain during exercise, 4: behaviour during exercise, 5: muscular symmetry, 6: performance/functional capacity, 7: behaviour at rest, 8: palpation, 9: balance and 10: proprioception.

Using experts’ experience and opinion, this study aimed to develop a consensus on the domains to be included in a model for a composite outcome score for horses undergoing rehabilitation. These data indicate that observational data (e.g., lameness and behaviour due to pain) and hands-on (e.g., palpation on soft tissue) were considered essential for inclusion within a musculoskeletal OM. The broad range of domains in this study’s model suggests that an outcome score needs to contain a variety of data. Indeed, this approach would provide a holistic view of the status of the horse undergoing therapy (Figure 2). 

### 4.1. Lameness

The highest agreement across the panel was for the inclusion of a lameness measurement within TEMROS. In equine practice lameness is typically evaluated by observing movement asymmetry in trot; however, this often presents a challenge, especially in horses presenting with low grade lameness [41,42]. For gold standard detection and evaluation, force plates are recommended, although these are not used outside the research environment and not practical for clinical assessment. Therefore, inertial sensor systems are useful where force plate analysis is not practical [43]. In practice, lameness assessment is commonly conducted by a visual gait assessment without technological equipment [41] and visual assessment, without technological equipment, has been investigated for both intra- and inter-rater reliability. Keegan et al. [42] studied the reliability of overground evaluation of lameness to determine if clinicians could agree on whether horses were lame and if so, which was the limb and score for the maximum level of lameness [42]. The American Association of Equine Practitioner (AAEP) scoring method was used, which is a 6-point scale where 0: Lameness not perceptible under any circumstances; 1: Lameness is difficult to observe and is not consistently apparent, regardless of circumstances (e.g., under saddle, circling, inclines, hard surface, etc.); 2: Lameness is difficult to observe at a walk or when trotting in a straight line but consistently apparent under certain circumstances (e.g., weight-carrying, circling, inclines, hard surface, etc.); 3: Lameness is consistently observable at a trot under all circumstances; 4: Lameness is obvious at a walk and 5: Lameness produces minimal weight bearing in motion and/or at rest or a complete inability to move. Keegan and colleagues [42] found that agreement of grading mild lameness was low (61.9%), although the agreement of lameness being present in horses scored greater than 1.5 on the AAEP scale was higher (93.1%) [42]. In addition, previous studies have shown lower agreement when practitioners assessed videos of lame horses [44,45,46]. Therefore, it is suggested that multiple evaluators should not be used to evaluate lameness. In contrast to the AAEP score, one prominent equine veterinarian reported that too many horses with different levels of lameness have to be graded 3 on the AAEP scale and therefore, in practice, they use their own scale [47,48]. This recommended scale has nine categories, where 0 = sound; 2 = mild; 4 = moderate; 6 = severe; 8 = non-weight bearing. The marked difference is that the grading system is applied in individual gaits and tests; for instance, in a straight line or on a circle, to give a more accurate picture of the lameness, as it is their consensus that 0–5 represents insufficient grades and other systems using scores 0–10 consistent of too many options to be useable [47]. Whilst lameness was the domain which achieved universal agreement (100%), hence it should be included, how lameness evaluation is integrated remains challenging especially in the presence of bilateral lameness, lameness occurring only with specific conditions such as under saddle or in the case of an asymmetric gait that is due to morphology or laterality. The premise of an outcome score for practitioners is that it should be easy to use in clinical practice; therefore, although technology may be increasingly available [49] whilst it is not yet in every practice or available to non-veterinary practitioners, a categorical subjective score would need to be included in TEMROS. The exact choice of grading system requires further study due to the absence of a universally accepted method that is easy to define, repeatable and can take into account the range of clinical presentations of lameness [47]. Until this is available, physiotherapists should evaluate lameness individually based on intra-rater reliability of lameness assessment being more reliable that inter-rater and that agreement between ‘improvement’ or ‘worsening’ in horses seen on multiple occasions is repeatable to use as an indicator of improvement, irrespective of the absolute score [46].

### 4.2. Pain Assessment

Four domains selected related to the assessment of pain: pain at rest; behaviour at rest; pain during exercise and behaviour during exercise. Whilst crucial to horse welfare, the recognition and measurement of pain in horses is widely acknowledged to be difficult [50,51] due to pain levels reported by an observer being subjective and open to bias [52]. Pain has been reported to change facial expression in mice [53], rats [54] and more recently, in horses via the horse grimace scale [50] and the equine pain face [51]. Both these equine scales have been validated for recording pain at rest by categorical scoring of facial expression and thus, either could be used for the pain and behaviour at rest domains within TEMROS. The use of pain assessment for chronic, longer term pain conditions would have to be considered in the context of rehabilitation as this process takes longer than the duration of pain evaluation in the trials. These scoring systems have been shown to have acceptable inter-rater reliability for horses with acute pain. It would be of interest to know if veterinary professionals score similarly to the non-trained carers of horses undergoing treatment. Whether carers can objectively evaluate pain and not be altered by bias in either direction has not been reported; nevertheless, it is important to ensure that accurate pain assessment leads to optimal pain management throughout the whole course of treatment. 

Pain and behaviour during exercise could theoretically be integrated within TEMROS via scoring of facial expressions [55] and whole-horse behaviours during in hand and groundwork, and ridden work [56]. The level of activity that the horse was undertaking at the stage of rehabilitation would have to be factored into the outcome score, as early phase programmes may prohibit ridden activity, so pain and behaviour during handling tasks such as leading or ground work would need to be considered. As well as the task and the environment the assessment occurs in, an additional element that may alter horse’s behaviour is the effect of the handler [57]. Therefore, the validity of pain assessment via facial expressions or whole horse behaviours during in-hand and groundwork with a handler and in different locations such as an indoor arena or an outside location needs to be studied further.

It is of significant importance to horse welfare that the signs of pain in horses, whether in the stable or whilst being handled/ridden are considered during assessment. Evaluation of rehabilitation progress would not be holistic without including monitoring of pain; therefore, further studies are required to test the application of pain assessment methods (e.g., equine pain face [51] or the ethogram for the assessment of pain in ridden horses [56]), specifically to rehabilitation programmes.

### 4.3. Muscle Symmetry

The need to evaluate muscle symmetry is apparent when considering pathologies such as those in the region of the sacro-iliac joint, which may result in asymmetric atrophy of the overlying gluteus medius muscle [58]. Thoracolumbosacral pain can result in thoracic epaxial muscle wastage [7,59] which anecdotally may be lateralised and therefore, asymmetric. Epaxial muscle size can be measured with ultrasound imaging [8,60,61] but this method may not always be accessible due to cost and its setting in veterinary or research laboratories. External muscle profile shape can be recorded with a low cost piece of equipment called a flexicurve ruler and this has been shown to be repeatable in the thoracic region [31]; however, the use of a flexicurve has not been reported on in other areas of the muscular system. The repeatability of a muscle scoring system devised by the authors of a study to investigate the relationship between thoracolumbar kinematics and muscle tone and tension in dressage horses found moderate agreement between five assessors (0.60–0.79) [62]. It was suggested that the muscle score could be used by physiotherapists to identify and monitor muscle development; however, the authors’ note the scale was subjective and only applicable to dressage horses. Therefore, if this domain is to be included within TEMROS objective measures need to be further developed for clinical practice and tested for reliability and validity for horses in all equestrian disciplines, to be applicable to the possible range of horses undergoing rehabilitation.

### 4.4. Performance/Functional Capacity

Most tests of performance in horses have a strong physiological basis, such as standard exercise tests, which evaluate relative speed and heart rate or blood lactate levels [63,64]. The intensity of the exercise effort in standard exercise tests, albeit submaximal, may not be appropriate for horses undergoing rehabilitation. A test of performance and functional capacity would need to be at lower exercise intensities and personalised to the stage of rehabilitation [65,66]. In human sports, medicine function performance tests are used to evaluate return to play status in footballers [67], muscle strength and functional performance in recreational athletes following anterior cruciate ligament reconstruction [68] as well as function in patients with patella tendinosis or achilles tendinopathy [20,21]. Similarly, in dogs, functional tests are available such as the Canine Brief Pain Index and the Helsinki Chronic Pain Index [23,54] which include questions on tasks such as how well the dog rises to standing and willingness to walk or run. A functional score for dogs with neurological conditions has been tested for inter-rater reliability by seven observers scoring tasks of progressive difficulty such as standing up from lying, walking in turns or walking stairs [25]. The performance was graded with a numeric score from 0, indicating that the dog cannot perform the task to 4, which represented normal motor function. No such scores exist in equine assessment but a simple battery of tests could be devised that included movements such as flexion of the neck [69] and turning small circles [70]. Any such testing procedure would need to be subject to evaluation of face and content validity and reliability testing similar to the neurological function tests for dogs devised by Boström et al. [25].

### 4.5. Palpation

The panel agreed that palpation should be included in the proposed composite outcome score and it was expected that manual palpation would be required as local assessment of soft tissues and joint margins is commonly undertaken when assessing injury and pain [70]. Response to manual palpation can be evaluated in the form of the behavioural response and/or evaluation of localised short-term change in the tissue being palpated, with a lower threshold to the onset of these responses indicative of a higher level of pain arising from these soft tissues [26,27,28,71,72]. Pain sensitivity, as a subjective experience, is individually variable in humans and based on complex physical and psychological interactions [73]; similarly, third-party assessment of pain in animals has found wide intra-species variation exists as well as reported differences between species [74]. In horses, subjective judgement of pain thresholds by manual palpation is commonplace [28]; therefore, the use of quantitative tools to assess responses to palpation may be preferable to subjective pain assessment because this allows rating of response with a force output. Pressure algometry (PA) uses a calibrated pressure gauge to objectively record the threshold the onset of pain in the tissues it is applied on [71]. The PA has been used to evaluate chiropractic interventions for equine thoracolumbar pain [11] and algometry measurements correlate with palpation scores (*r* =−0.90) where the threshold for onset of pain increases as pain reduces [28]. However, reports that repeated PA application can result in sensitivity or habituation to the PA tool [29,71] could limit their validity in clinical practice. As an alternative, categorical scoring systems can be used to score response to manual palpation and use of this form of reporting could be integrated into TEMROS [9,27,28,29]. Merrifield-Jones et al. [29] used a six-point score, where 0 is described as soft, low tone; 1 as normal; 2 as increased muscle tone but painful; 3 as increased muscle tone and/or painful (slight associated spasm on palpation, no associated movement; 4 painful (associated spasm on palpation with associated local movement, i.e., pelvis tilt, extension response) and 5 as very painful (spasm plus behavioural response to palpation, i.e., ears flat back, kicking). This score has shown excellent inter-rater reliability on a small sample of ten riding school horses between three physiotherapists when assessing epaxial soft tissue (ICC 0.09) [29]. The use of the PA tool, if practitioners were trained, could provide objective data if habituation and sensitisation were considered but the use of a categorical scale would provide a cost effective and convenient method of assessing response to palpation.

### 4.6. Balance and Proprioception

The final two domains that reached the minimal critical value for inclusion were balance and proprioception. The first study to measure balance in horses investigated postural sway using force platforms demonstrated that the standing horse has small movements of the centre of pressure resulting from small adjustments of muscle tension, indicating the stability of the quiet standing horse’s centre of mass [75]. Whilst balance has not been measured in relation to musculoskeletal injury, motion of the centre of pressure does increase with medical sedation administered intravenously [76]. Signs of ataxia, such as trembling, locking and unlocking of joints, weight shifts and obvious swaying, were observed and it could be theorized that injury to one component required to maintain balance, such as sensory input, motor responses and cognitive processes [75], could have similar effects. To further examine potential clinical signs from neurological deficits, in relation to balance, twenty horses were blindfolded whilst stood on a force platform [77]. In these horses, movement amount and velocity increased, and showed greater within-trial variability, when horses were blindfolded compared to their sighted measurements. Force platforms have been used as a primary outcome variable to assess the effects of osteoarthritis, surgically induced into the carpal joint in a group of 16 young horses [78]. Half of this cohort underwent an exercise regime on a water treadmill from 15 days following the surgery, five days a week for a total on ten weeks. At reassessment, the horses that had been exercised on the water treadmill had significantly improved static balance control compared to control group of horses with carpal joint osteoarthritis. It should be noted that whilst these three force plate studies assessed postural sway during stance, gait involves spinal reflexes that might respond differently to effect balance during locomotion [76]; therefore, the results are limited as they cannot directly be translated to balance during gait.

Proprioception, as a domain listed to be included in TEMROS, does not have any objective measurement techniques reported for horses. However, postural stability relies on motor components of the musculoskeletal system to maintain balance and this includes proprioceptive information. Muscles induce joint motion and are also responsible to stabilising joints during motion therefore proprioceptive feedback is crucial to balance control [75,78]. Impairment to sensory and motor components, possibly due to joint injury, could affect postural control and if measured could also provide a proxy for proprioceptive deficit, but understanding this relationship within the scope of equine rehabilitation requires further analysis. 

Force platforms could be used to measure balance and proprioceptive changes as a result of therapeutic interventions, although laboratory-based equipment is required because equine force platforms are not easily mobile. For clinical practice other methods to measure balance are necessary. Exercises to challenge balance and activate the trunk core muscles have been suggested as part of rehabilitation plans [79]. These exercises destabilise the horse by lifting a limb and inducing a weight transference to the contra- or ipsi-lateral weight-bearing limbs, however they do not have any form measurement to evaluate their effectiveness. A pressure mat that measures percentage weight distribution between limbs is available for canine orthopaedic assessment [80] and if a similar measurement method or a score system could be developed for horses then these positions could be used as a form of balance evaluation. 

### 4.7. Limitations to the Study

The number of experts selected to participate was small and was carried out based on the criteria (knowledge of research published and industry expertise) of the authors. This could present bias to the panel however once formed, TEMROS could be presented to the wider equine community for consideration and content validation. It would have been of benefit to have an understanding of the rationale for inclusion [81] to allow retrospective analysis of domains chosen. The high levels of agreement for the domains selected supports the consensus is based on common experience and practice. 

Although a wide literature search has been completed to map potential reliable and valid measurement tools/tests to each domain it is possible that there are suitable tests/tools which were not suggested for inclusion by the panel. An example is thermography which has been used to measure surface temperature of racehorses’ epaxial muscles in response to training [82]. Skin temperature measurements have not been used to evaluate effects of rehabilitation intervention however the reducing cost of thermography cameras may allow more horses to be imaged with this non-invasive and non-ionizing modality, albeit following strict protocols for carrying out and analysing results [83]. It should be noted that the choices of tests are evaluated in relation to those considered practical and feasible to use ex vivo. To be valid as a measure of rehabilitation outcome, each domain should have face validity which is a key factor in the development of an efficient OM is for the score in the absence of any gold standard [13].

### 4.8. TEMROS—Further Development

A composite score integrating the above domains takes into account several behaviours and physiological parameters by including scores for each specific parameter. There are domains that have various scoring systems or measurement tools, such as lameness and palpation and the final system/tool which require further testing to be validated. There are also domains where measurement techniques have yet to be designed for or tested, for instance muscle symmetry and proprioception, and therefore these areas need further development. Some of the parameters could be weighted according to perceived significance or they could be graded equally [33] and evaluation of this requires further development. However, TEMROS has the potential to provide a holistic assessment which would be relevant to rehabilitation of injury, as the whole horse is undergoing the rehabilitation not just the condition.

## 5. Conclusions

The Delphi methodology was successfully applied to attain consensus across the selected international expert panel that there is a need for an outcome measure for equine rehabilitation and agreement on the domains that such a measure should include. The expert panel agreed that lameness, pain at rest, pain during exercise, behaviour during exercise, muscular symmetry, performance/functional capacity, behaviour at rest, palpation, balance and proprioception should be included. The challenge going forward is to combine measures for each of these domains that are reliable, valid and easy to use in clinical practice. With reliably measured domains, and subsequent validity testing, TEMROS could provide a composite score with equine practitioner consensus that could support clinical practice as well as substantiate treatment choices to improve horse welfare.
